# A Four-Channel Low-Noise Readout IC for Air Flow Measurement Using Hot Wire Anemometer in 0.18 μm CMOS Technology

**DOI:** 10.3390/s21144694

**Published:** 2021-07-09

**Authors:** Kyeongsik Nam, Hyungseup Kim, Yongsu Kwon, Gyuri Choi, Taeyup Kim, Chulhong Kim, Dongil Cho, Junghoon Lee, Hyoungho Ko

**Affiliations:** 1 Department of Electronics Engineering, Chungnam National University, Daejeon 34134, Korea; ksnam@o.cnu.ac.kr (K.N.); hyungseup@cnu.ac.kr (H.K.); yongmayer@cnu.ac.kr (Y.K.); cyeakl@o.cnu.ac.kr (G.C.); 2 Department of Electrical and Computer Engineering, Automation System Research Institute (ASRI), Inter-University Semiconductor Research Center (ISRC), Seoul National University, Seoul 08826, Korea; taeyupk@snu.ac.kr (T.K.); chkim89@snu.ac.kr (C.K.); dicho@snu.ac.kr (D.C.); 3 Department of Mechanical and Aerospace Engineering, Seoul National University, Seoul 08826, Korea; jleenano@snu.ac.kr

**Keywords:** automatic offset calibration loop (AOCL), instrumentation amplifier (IA), readout integrated circuit, air flow measurement

## Abstract

Air flow measurements provide significant information required for understanding the characteristics of insect movement. This study proposes a four-channel low-noise readout integrated circuit (IC) in order to measure air flow (air velocity), which can be beneficial to insect biomimetic robot systems that have been studied recently. Instrumentation amplifiers (IAs) with low-noise characteristics in readout ICs are essential because the air flow of an insect’s movement, which is electrically converted using a microelectromechanical systems (MEMS) sensor, generally produces a small signal. The fundamental architecture employed in the readout IC is a three op amp IA, and it accomplishes low-noise characteristics by chopping. Moreover, the readout IC has a four-channel input structure and implements an automatic offset calibration loop (AOCL) for input offset correction. The AOCL based on the binary search logic adjusts the output offset by controlling the input voltage bias generated by the R-2R digital-to-analog converter (DAC). The electrically converted air flow signal is amplified using a three op amp IA, which is passed through a low-pass filter (LPF) for ripple rejection that is generated by chopping, and converted to a digital code by a 12-bit successive approximation register (SAR) analog-to-digital converter (ADC). Furthermore, the readout IC contains a low-dropout (LDO) regulator that enables the supply voltage to drive digital circuits, and a serial peripheral interface (SPI) for digital communication. The readout IC is designed with a 0.18 μm CMOS process and the current consumption is 1.886 mA at 3.3 V supply voltage. The IC has an active area of 6.78 mm^2^ and input-referred noise (IRN) characteristics of 95.4 nV/√Hz at 1 Hz.

## 1. Introduction

Recently, the concept of learning from nature has rapidly spread across several scientific communities, such as computer science, engineering, and robotics. Learning from nature, such as mimicking the movement of insects, is separated into several stages, which include the data sensing stage. In this stage, there are numerous types of microelectromechanical systems (MEMS) sensors, such as acceleration [1,2], position [3], temperature [4,5,6], and humidity sensors [7,8]. The main objective was to measure movements of insects, such as flying. An insect in flight is substantially modulated by the airflow simulation of a head wind [9,10]. Such airflow sensing technology can be actively applied to the insect biomimetic robots field. The biomimetic robot system enables the condition monitoring and remote management of the insect robot’s movement [11,12]. Therefore, MEMS sensors and readout integrated circuits (ICs) for condition monitoring and converting the output signals of the sensors into electrical signals are being actively researched, respectively [13]. In an insect’s movement, it is crucial to monitor the conditions surrounding the air flow. Variable information such as the air velocity, air acceleration, humidity, temperature, and atmospheric pressure can be obtained by measuring the air flow in the atmosphere [14,15].

**Figure 1:**
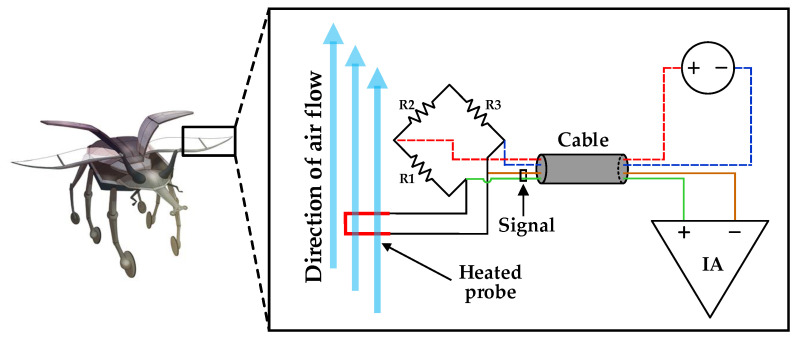
Hot wire anemometer structure for modeling insect’s wing and measuring airflow.

Air flow is generally measured by examining the hot wire anemometer. The conventional hot wire anemometer structure is illustrated in Figure 1. A hot wire anemometer works when an electrically heated wire is placed in a flowing air stream; consequently, heat is transferred from the wire to the air, which triggers a reduction in the temperature of the wire [16,17] and alters the resistance of the wire. This change in the resistance of the wire is a measure of the flow rate, flow velocity, and flow acceleration [18,19].

The micro anemometer probe of the air flow sensor assesses the resistance variation of the hot wire anemometer and generally converts the measured temperature data into resistance. The sensor that converts input data into resistance is termed a resistive sensor and has a comparatively higher reliability and simpler structure than other types of sensors [20,21,22]. Consequently, resistive sensors are ideal for biomimetic robot systems, and they require accurate production. However, existing research requires an impedance analyzer to convert the converted resistance into a digital signal capable of signal processing and data communication. In this study, low-noise, low-power, and miniaturization characteristics were realized by superseding a commercial impedance analyzer using a resistive readout IC.

The resistive readout IC converts the input resistance change into a voltage alteration by employing diverse structures such as the Wheatstone bridge at the input stage. Due to the fact that the voltage alterations typically have small values, they are adequately amplified using an instrumentation amplifier (IA), and then converted into digital signals by utilizing an analog-to-digital converter (ADC). Therefore, it is important to design an IA with a high linearity and low noise. There are three types of IAs in resistive readout ICs, each having its own advantages and disadvantages.

The first is a capacitively coupled IA (CCIA) with an input stage configured as a capacitor. The conventional structure of the CCIA is advantageous in terms of power efficiency because it contains only one op amp. However, because the input stage comprises a capacitor, it is limited, as the input impedance is smaller than that of the IA of the other structures. To address this limitation, an additional circuit such as a positive feedback loop (PFL), which boosts the input impedance, is required [23].

The second IA architecture is a current feedback IA (CFIA) that feeds back the current. The CFIA has the advantage of a high input impedance because the input stage comprises the gate of a MOSFET in the op amp. However, if there is a mismatch between the input transconductance and the feedback transconductance, the linearity and gain precision of the circuit are restricted [23].

The last IA architecture is a three op amp IA structure consisting of two amplification stages with three op amps. For a three op amp IA to realize low-noise characteristics, two op amps connected to the input stage should have low-noise characteristics. In general, the low-noise op amp consumes a significant amount of current, such that the three op amp IA structure has a lower power efficiency than that of the CCIA. However, as with the CFIA, the input stage is composed of MOSFET gates, such that the input impedance and linearity are higher than those of the CCIA. Furthermore, because the gain stage is composed of two stages, gain tuning is comparatively simple, and the range of gain is wider than that of CCIA and CFIA. Consequently, the three op amp IA structure is primarily implemented in commercial impedance analyzers. This structure was also employed in the resistive readout IC in this study [24,25].

In addition, when the micro anemometer probe performs the role of a heated probe in the hot wire anemometer structure, heating is required by applying a current to the air flow sensor. To accomplish this, a current generator is included in the input stage of the resistive readout IC. The three-op amp IA comprises a chopper at the input and output stages, and it employs a 4th-order low-pass filter (LPF) in order to eliminate high-frequency components. The signal from which the high-frequency component is removed through the 4th-order LPF is converted into a digital signal through a 12-bit successive approximation register (SAR) analog-to-digital converter (ADC).

In other words, the proposed resistive readout IC comprises a current generator, 4th-order LPF, and 12-bit SAR ADC, and it contains an auto-offset calibration loop (AOCL) to automatically calibrate the input offset. The AOCL corrects the offset of the input stage by employing an R-2R digital-to-analog converter (DAC) while binary searching the output voltage of the 4th-order LPF. A serial peripheral interface (SPI) is adopted for fundamental communication, and a low-dropout (LDO) regulator is also built into the same chip to furnish the supply voltage for these digital circuits. In this study, because the proposed four-channel low-noise readout IC exhibits a reconfigurable gain, it can measure a wide range of input resistances. Furthermore, the readout IC exhibits a high precision and linearity by implementing a low-noise three-op amp IA architecture.

The rest of this paper is organized as follows. Section 2 describes the structure and operating principle of the micro anemometer air flow sensor and the four-channel low-noise readout IC. Section 3 presents the measurement results of the fabricated airflow sensor and readout IC. Finally, Section 4 concludes the paper with a performance summary and comparison.

## 2. Proposed Micro Anemometer Air Flow Sensor and Low-Noise Readout IC

This section discusses the micro anemometer air flow sensor and low-noise readout IC proposed in this study. Section 2.1 describes the structure and operating principle of the micro anemometer air flow sensor. Section 2.2 comprehensively discusses the architecture and operating principle of low-noise readout ICs and sub-blocks.

### 2.1. Micro Anemometer Air Flow Sensor

**Figure 2:**
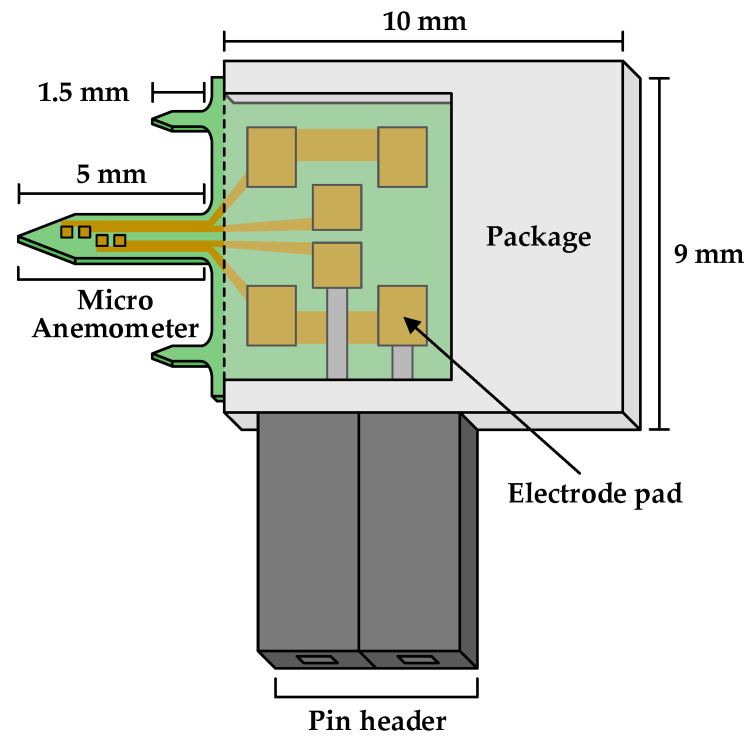
Structure of the proposed micro anemometer air flow sensor.

The structure of the micro anemometer air flow sensor proposed in this study is shown in Figure 2. The proposed air flow sensor is largely divided into an anemometer part and an electrode pad part, and the anemometer is measured by a micro anemometer. The anemometer adopted is 5 mm long, 500 µm wide, and 300 µm thick.

There are four patterns in the micro anemometer, comprising two resistors each: up and down. When the air flows into this pattern, the temperature of the pattern changes according to the amount of air flow; hence, the resistance formed by the pattern also changes. At this point, the default value of the resistance was 100 Ω, and the change in the resistance was approximately ±1% (±1 Ω). This resistance was converted into a digital signal using the proposed readout IC.

**Figure 3:**
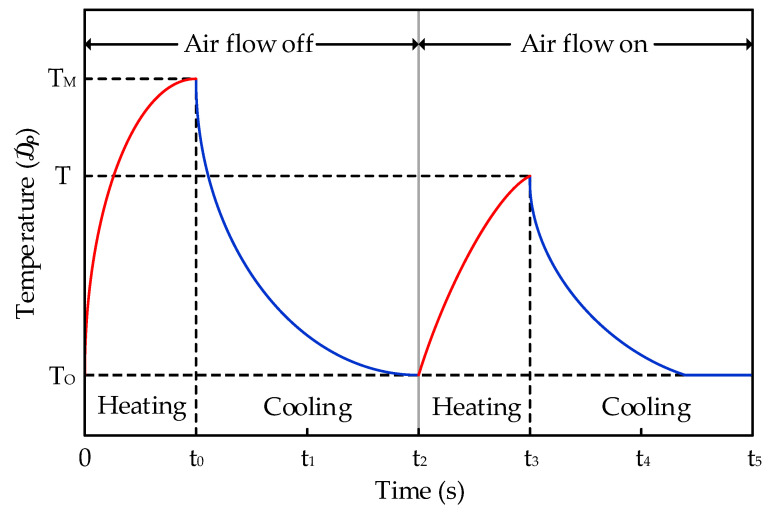
Operation principle of the micro anemometer air flow sensor.

The operation principle of the proposed micro anemometer is shown in Figure 3. The peak temperature at the end of the heating period is reduced when the air flow exists.

When the sensor is heating and cooling, it can be assumed that the input amount of heat by the Joule effect in a constant air flow is the same as the amount of heat emitted, which can be written as

(1) $$h\cdot S(T-T_{O})=R\cdot I^{2}$$

where *h*: coefficient of heat exchange (W∙m^−2^∙°C^−1^); *S*: exchange surface area (m^2^); *R*: electrical resistance of heating element (Ω); *I*: intensity of electric current (A); *T_O_*: temperature of micro anemometer when not heating (°C); *T_M_*: temperature of micro anemometer in absence of air flow (°C); *T*: temperature of micro anemometer when there is air flow (°C).

If *h_O_* is designated the coefficient of heat exchange in the absence of air flow and h is assumed to be related to the air flux density (air flow velocity) u, then h is given by

(2) $$h=h_{O}(1+\alpha \cdot u)$$

If there is no air flow (*u* = 0), then *h_O_* and *h* are equal, and *h_O_* can be expressed as

(3) $$h_{O}=h=\frac{R\cdot I^{2}}{S(T_{M}-T_{O})}$$

Furthermore, summarizing (2) with respect to *u*, the equation can be written as

(4) $$u=\frac{1}{\alpha }\cdot \left(\frac{h-h_{O}}{h_{O}}\right)=\frac{1}{\alpha }\cdot \left(\frac{T_{M}-T}{T-T_{O}}\right)$$

Consequently, the total air flow *F* can be calculated as the product of *u* and surface area *S_a_*:

(5) $$F=u\cdot S_{a}$$

In Equation (4), *a* is the empirical constant that can be acquired through experiments, and *T_M_*, *T*, and TO are the temperatures of the micro anemometer in each environment. Therefore, the air flux density u can be calculated by evaluating this temperature. The air flow sensor proposed in this paper converts temperature data from the micro anemometer into resistance and then converts it to a digital signal using the low-noise IC presented in Section 2.2.

### 2.2. Four-Channel Low-Noise Readout IC

**Figure 4:**
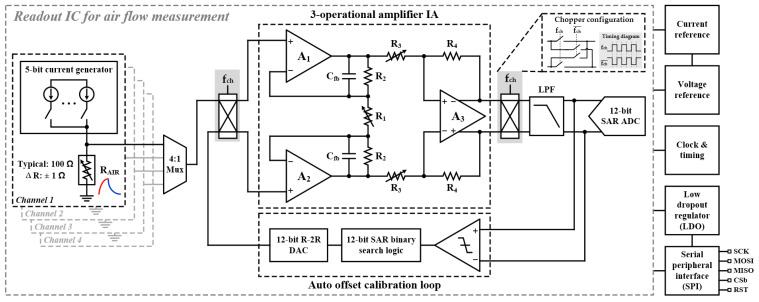
Top architecture of proposed four-channel low-noise readout IC for air flow measurement.

The four-channel low-noise readout IC is shown in Figure 4. The input stage of the proposed circuit comprises a current generator that applies current to the resistor (*R_AIR_*) formed in the micro anemometer air flow sensor and a 4:1 mux to the selection of the desired channel among the four channels. The current generator consists of 5 bits, and the output current can be altered in 32 steps in total. This output current flows to an external resistor *R_AIR_*, and the input voltage is determined by the product of the output current and the resistance of the *R_AIR_*. The input voltage signal is amplified using a three op amp IA. The three op amp IA comprises a first stage consisting of *A*_1_, *A*_2_, *C_fb_*, *R*_1_, and *R*_2_, and a second stage consisting of *A*_3_, *R*_3_, and *R*_4_. The transfer function of the three op amp IA is calculated as

(6) $$\frac{V_{out}}{V_{in}}=\frac{R_{1}+2R_{2}+sR_{1}\cdot R_{2}\cdot C_{fb}}{R_{1}+sR_{1}\cdot R_{2}\cdot C_{fb}}\cdot \frac{R_{4}}{R_{3}}$$

Because the air flow measurement method proposed in this study has a very slow input signal (s = jw = 0), (6) is simplified as

(7) $$\frac{V_{out}}{V_{in}}=\left(1+\frac{2R_{2}}{R_{1}}\right)\cdot \frac{R_{4}}{R_{3}}$$

According to (7), the overall gain can be altered by adjusting the *R*_1_ and *R*_3_ of the three op amp IA. In the proposed circuit, *R*_1_ and *R*_3_ are designed to have a wide range of gains by implementing 5-bit and 4-bit programmable resistors, respectively.

The proposed readout IC is designed to realize low-noise by employing a chopper in the input and output stages of the three op amp IA. The low frequency noise of the current generator can be reduced by the on-and-off (chopping) operation of the current generator; however, the current generator is enabled when heating and is disabled when cooling, so the chopper operation is hard to apply to the current generator. Therefore, in this design, we implemented the choppers on the three op amp IA’s input and output for reducing the flicker noise.

The input signal that passes through the 4:1 mux is modulated into the high-frequency region by the input chopper and demodulated back into the low-frequency region by the output chopper. In contrast, the offsets present in *V_inp_* and *V_inn_* are unaffected by the input chopper and are only modulated in the high-frequency range by the output chopper. Consequently, the low-frequency input signal and high-frequency modulated offset component remain at the output stage of the three-op amp IA, and a 4th-order LPF is implemented in order to eliminate the high-frequency offset component. The signal that has passed through the LPF is converted to a digital signal using a 12-bit SAR ADC.

In Figure 4, *V_inp_* of the input voltage is determined by the output current of the current generator and resistance *R_AIR_*. However, *V_inn_* should be determined to be the same voltage as the offset of *V_inp_*, which is realized by the AOCL. If there is an offset of *V_inp_* and *V_inn_*, a difference in the output voltage of the LPF occurs. After carrying out comparisons using a comparator, the offset is calibrated using the binary search process and R-2R DAC.

In addition, the proposed readout IC includes a bias block, LDO regulator, and clock generator for the operation of the SAR ADC. The digital signal is finally output through the 12-bit SAR ADC and is sent externally through the master input slave output (MISO) of the SPI. The various registers of the readout IC can also be controlled by the SPI. Although the analog circuit applies a 3.3 V supply voltage, digital circuits such as the SPI employ a 1.8 V supply voltage; hence, an external power supply or DC-DC converter is required. When external power is applied, the test board structure can be complicated and influenced by external noise; therefore, an LDO regulator is added inside the same chip to prevent this phenomenon. The added LDO regulator converts 3.3 V to 1.8 V by employing a single op amp, which furnishes the supply voltage to the SPI and digital circuitry.

**Figure 5:**
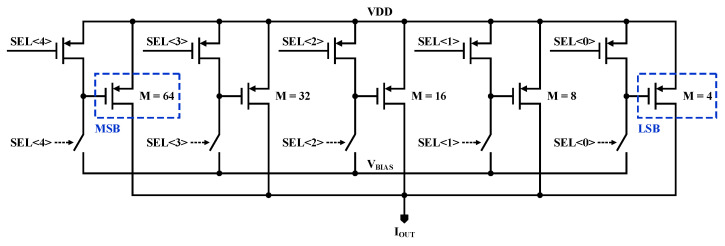
Schematic of proposed 5-bit current generator.

A schematic of the current of the current generator that induces the current in order to heat the *R_AIR_* resistor is shown in Figure 5. The current generator can adjust the output current to 5 bits, with each bit having a binary weight. There is a PMOS that generates current for each bit, and the output current is determined by the multiplier (*M*) of the PMOS and the *V_BIAS_* voltage, which is simplified as

(8) $$I_{OUT}=-\frac{1}{2}\mu_{p}\cdot C_{ox}\cdot \frac{(unit\;W)\cdot M}{L}\cdot {\left(V_{BIAS}-VDD-V_{thp}\right)}^{2}$$

The proposed current generator is designed such that the multiplier of the PMOS has a binary weight, and the PMOS corresponding to each bit can be turned on and off using the PMOS switch and CMOS switch.

**Figure 6:**
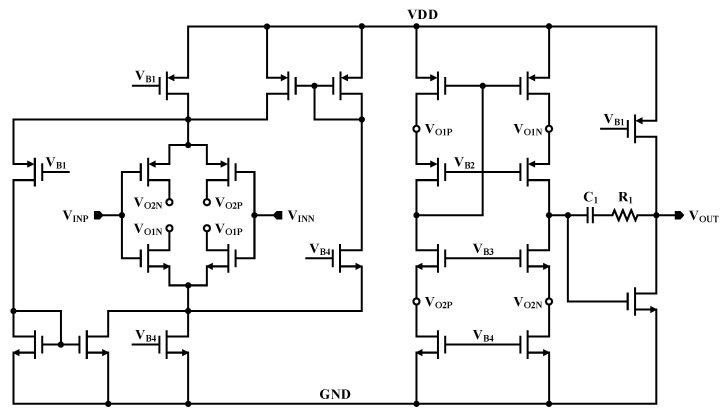
Schematic of proposed differential amplifier.

A schematic of the differential amplifier implemented in the first stage of three op amp IA is shown in Figure 6. The differential amplifiers with only the PMOS or NMOS input stages have a limited input voltage range, so the proposed differential amplifier employs a rail-to-rail input stage to surmount this drawback. Because the gain of the differential amplifier is related to the gm of the input stage, the nonlinearity of the gain increases when the gm is changed. To overcome this shortcoming, a constant-gm scheme that can sustain the gm of the entire input stage is applied. The intermediate stage of the amplifier adapts a folded cascode architecture in order to enhance the DC gain and unit bandwidth, and a class-A structure with an advantage in linearity is implemented as an output stage. Moreover, Miller compensation is performed utilizing a *C_1_* capacitor to ensure stability.

**Figure 7:**
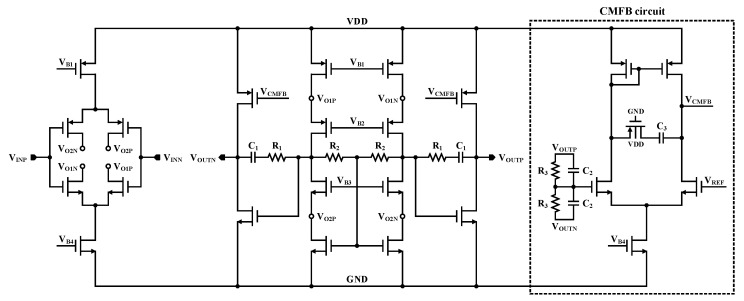
Schematic of the proposed fully differential amplifier.

A schematic of the fully differential amplifier implemented in the second stage of the three op amp IA is shown in Figure 7. The fundamental scheme of a fully differential amplifier is similar to that of a differential amplifier, and two steps of common-mode feedback (CMFB) are performed. The first is a resistive-CMFB architecture applied to *N*_1_ and *N*_2_ of the folded cascode stage. If the common-mode voltage of node A increases, then the gate voltages of *N*_1_ and *N*_2_ are augmented, and the current flowing through them also rises. The current increase reduces the common-mode voltage of *V_O2_* and results in negative feedback that declines the voltage of node A.

The second CMFB is an amplifier-CMFB architecture formed at the output stage. If the common-mode voltage of *V_OUT_* increases, then the voltage of node B also improves. That is, the gate voltage of *N*_3_ rises, and the flowing current is also augmented. As a result, the gate voltages of *P*_1_ and *P*_2_ diminish, and the *V_CMFB_* voltage increases. This increase in voltage eventually reduces the common-mode voltage of *V_OUT_*, resulting in negative feedback. This two-stage CMFB ensures a nearly constant common-mode output voltage and enhances the common-mode rejection ratio (CMRR).

**Figure 8:**
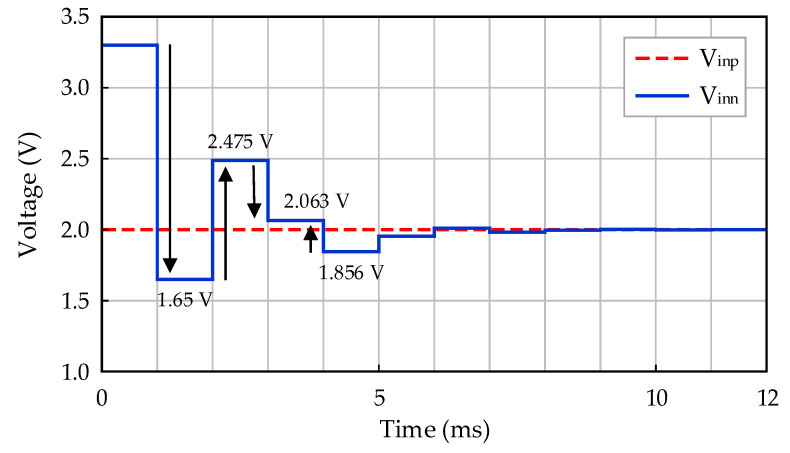
Operation example of AOCL.

The offset calibration process is required because the input signal offset cannot be accurately estimated. For this purpose, an AOCL architecture is implemented. An operation example of AOCL when *V_inp_* is 2 V is shown in Figure 8. In Figure 8, *V_inp_* does not change, and over time, *V_inn_* becomes nearly identical to *V_inp_*. *V_inn_* performs a binary search operation every 1 ms. *V_inn_* decreases when it is larger than *V_inp_* and increases when it is smaller than *V_inp_*. The magnitude of the increasing and decreasing voltage is half that of the previously increasing and decreasing magnitude. Because one cycle is 1 ms and 12-bit SAR logic and R-2R DAC are employed, the operation of the AOCL takes a total of 12 ms.

## 3. Experimental Methods and Results

**Figure 9:**
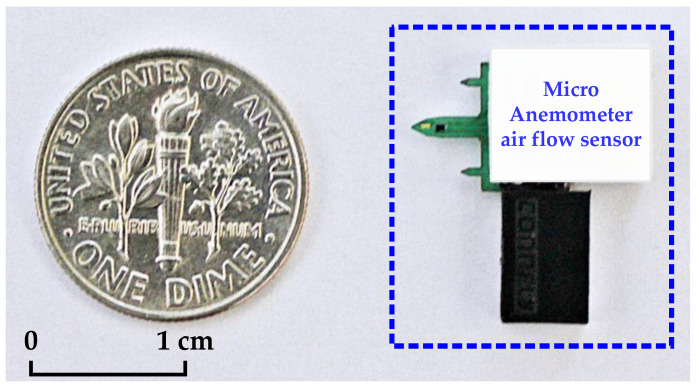
Photograph of manufactured micro anemometer air flow sensor.

**Figure 10:**
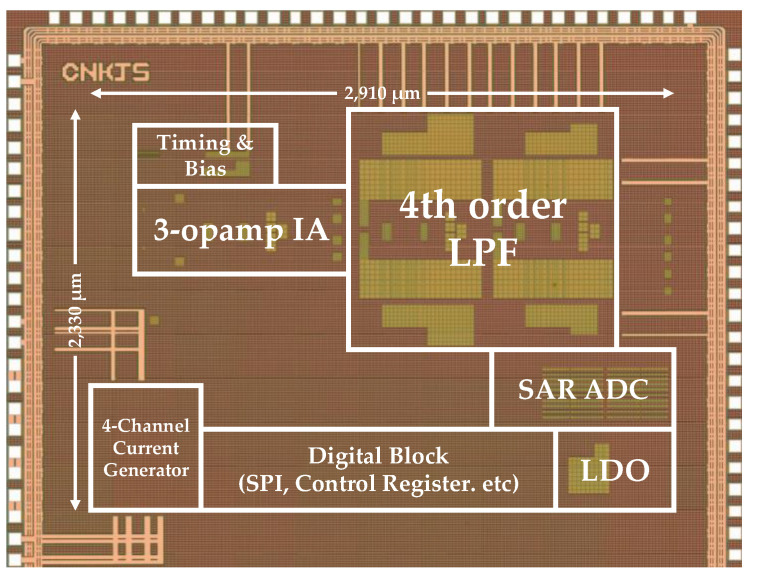
Die photograph of fabricated four-channel low-noise readout IC.

The fabricated air flow measurement sensor is shown in Figure 9, and Figure 10 presents the fabricated four-channel low-noise readout IC. The readout IC proposed in this study was fabricated using a 0.18 μm 1-poly 6-metal (1P6M) CMOS process and consumed 1.886 mA of current at a 3.3 V supply voltage. The active area of the fabricated chip was 6.78 mm^2^, and the readout IC for air flow measurement, current reference, voltage reference, timing generator, 12-bit SAR ADC, LDO, and SPI, were integrated into the chip.

**Figure 11:**
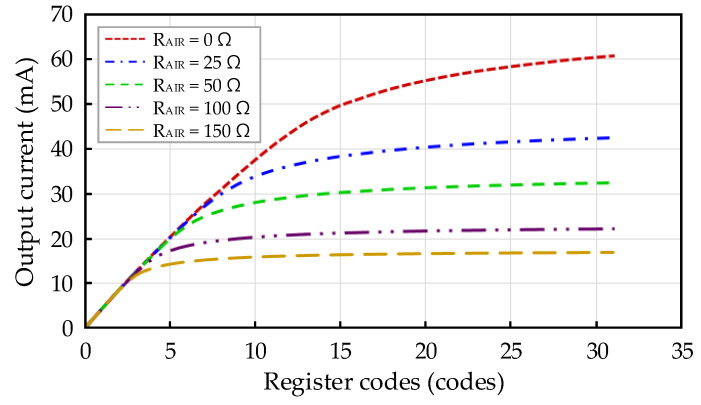
Output current measurement result of current generator based on load resistance.

The output current measurement results of the current generator in the four-channel low-noise readout IC are shown in Figure 11. The output current was measured by changing the external load resistor *R_AIR_* from 0 to 150 Ω. In addition, when *R_AIR_* had each value, the output current was confirmed by sweeping the 5-bit register of the current generator. If *R_AIR_* was the default value (100 Ω) and the register setting is the default setting (register codes: 8), then the output current was 19.65 mA. Consequently, the input voltage was 1.965 V. The default output current was designed for a simulation of 20 mA. Accordingly, the expected input voltage (*V_inp_*) was 2 V. The measurement results indicate that the output current is almost identical to that of the simulation results. The maximum output currents when *R_AIR_* is 100 Ω and 0 Ω were 22.23 mA and 60.62 mA, respectively.

**Figure 12:**
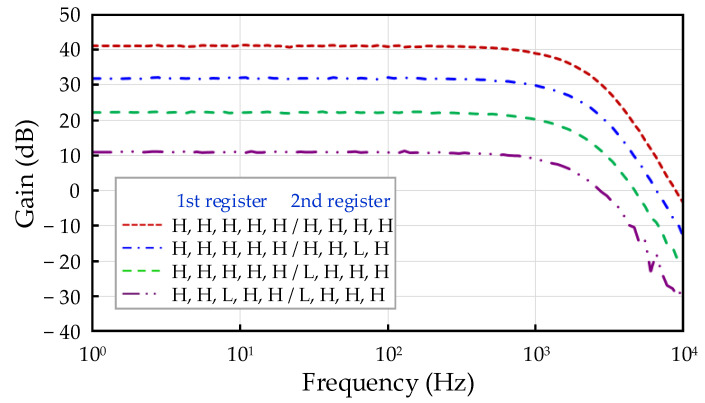
Transfer function measurement results based on register setting of readout IC.

The transfer function measurement results of the fabricated readout IC are shown in Figure 12. The frequency was swept using a dynamic signal analyzer, and the transfer function was measured between the input stage of the three op amp IA and the output stage of the 4th-order LPF. The designed three op amp IA comprised two amplification stages. The registers of the programmable resistors of the first and second stages were composed of 5 bits and 4 bits, respectively. The measurement indicated that the minimum and maximum gains of the three-op amp IA were 4.08 dB (1.60 times) and 47.47 dB (236.28 times), respectively. In addition, because the cutoff frequency of the 4th-order LPF was designed to be 1 kHz, the bandwidth of the readout IC was approximately 1 kHz. The four graphs in Figure 12 describe four levels of gain that change by approximately 10 dB, showing the register settings at the same time.

**Figure 13:**
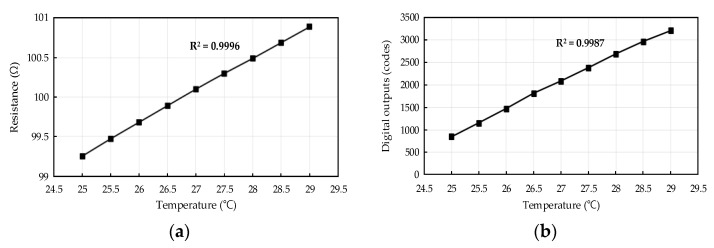
(**a**) Resistance measurement results based on temperature of manufactured air flow sensor; (**b**) digital output code measurement results based on temperature of fabricated four-channel low-noise readout IC.

The resistance measurement results of the air flow sensor based on the temperature, and the digital output code measurement result of the readout IC when receiving this resistance as input, are shown in Figure 13. The method of measurement is as follows. The micro anemometer air flow sensor is placed in a temperature chamber, and a readout IC is connected to the outside of the temperature chamber. Afterward, the temperature is altered. The air flow sensor had a resistance of 100 Ω at approximately 26.8 °C and a resistance change of ±1 Ω (±1%) in the range of approximately 25 °C to 29 °C. These resistance measurement results are shown in Figure 13a. Furthermore, if the gain of the readout IC that receives this resistance change is properly adjusted, then the digital output codes as shown in Figure 13b can be measured. Because the resolution of the SAR ADC in the readout IC was 12 bits, the output digital code range was 0–4095. Before starting the measurement, the AOCL was used when *R_AIR_* of the air flow sensor is closest to 100 Ω (27 °C) At this time, the digital output codes of the readout IC were approximately 2048. Then, the temperature is measured by changing the unit of 0.5 °C. It was seen that approximately 300 codes were altered when varying by 0.5 °C. The measurement results indicated that the *R*^2^ values of the air flow sensor and the readout IC were 0.9996 and 0.9987, respectively. Thus, the linearity of the sensor and circuit was secured.

**Figure 14:**
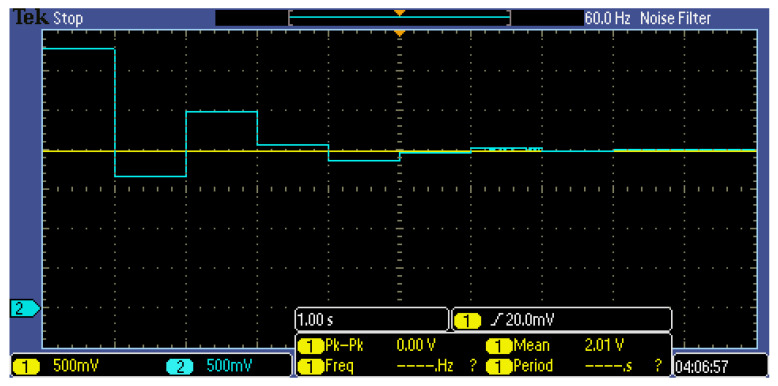
Measurement result of AOCL.

As a result of the AOCL operation measurement for correcting the input-stage offset of the readout IC, the output voltage (*V_inn_*) change in the AOCL is shown in Figure 14 when the input voltage (*V_inp_*) was 2 V. During measurements, the main clock of the AOCL was set to 1 Hz to explicitly confirm the operation, but the main clock of the default setting was 1 kHz, and the time required for offset calibration was 12 ms. The results of Figure 14 show that *V_inn_* followed the offset of *V_inp_* well, and a further measurement confirmed that the AOCL operated normally even when *V_inp_* was in the range of 0.1 V to 3.2 V.

**Figure 15:**
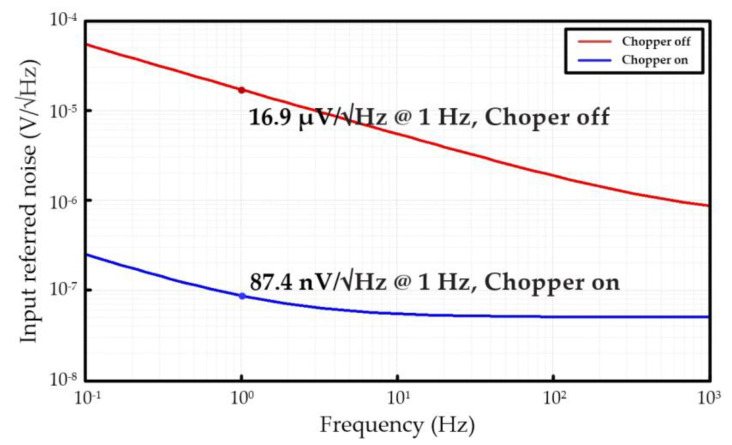
Input-referred noise simulation result of readout IC.

The input-referred noise (IRN) simulation of the readout IC is shown in Figure 15. The IRN at 1 Hz was reduced to 1/193 times from 16.9447 μV/√Hz without chopper operation to 87.4 nV/√Hz with chopper operation. The application of the chopper at the input and output stages of the IA can effectively reduce the IRN.

**Figure 16:**
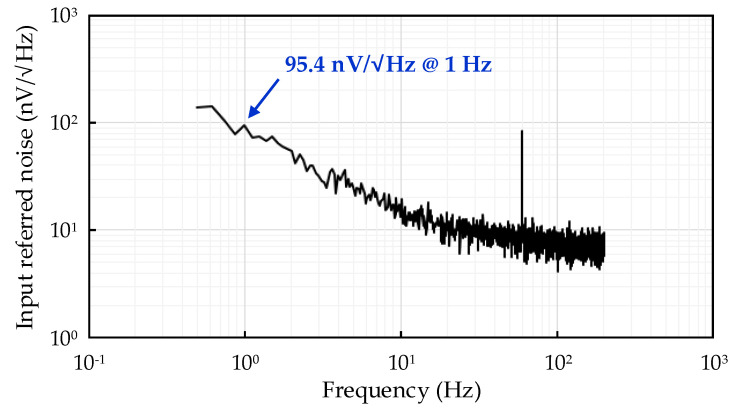
Input-referred noise measurement result of readout IC.

The IRN measurement of the fabricated readout IC is shown in Figure 16. Measurement of the IRN is performed by implementing a dynamic signal analyzer in the range of 0.5 Hz to 200 Hz. The simulated and measured IRNs were 87.4 nV√Hz and 95.4 nV/√Hz at 1 Hz, respectively.

**Figure 17:**
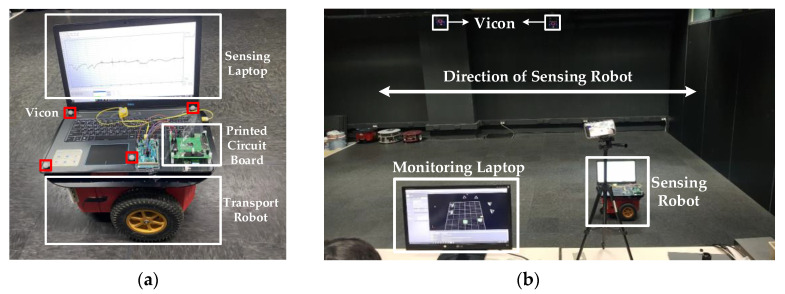
(**a**) Photograph of sensing robot for measuring air flow; (**b**) photograph of measurement environment.

The measurement environment and sensing robot settings for measuring the airflow velocity based on the time domain are shown in Figure 17. The sensing robot consists of a small printed circuit board (PCB) for the proposed readout IC and micro anemometer, a sensing laptop, four Vicons, and a transport robot. Four Vicons measurements were adopted in order to compare the data values of the proposed readout IC. The structure of the sensing robot is shown in Figure 17a. In this study, the air flow velocity was measured using the VVicons of the sensing robot moving in the directions of the *x*-axis and *y*-axis, and the data monitored in the monitoring laptop were compared with the data of the proposed readout IC. The measurement environment is shown in Figure 17b.

**Figure 18:**
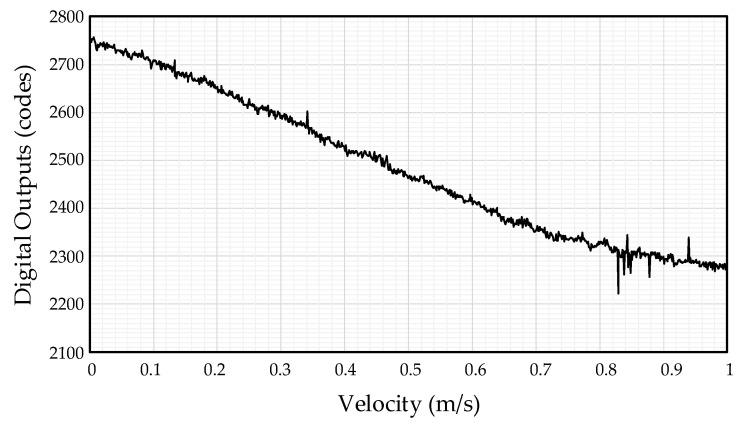
Digital code measurement result based on air flow velocity of readout IC.

The velocity measurement with digital code output of the proposed air flow sensor is shown in Figure 18. Measurements of the digital codes were performed by a 12-bit SAR ADC in the range of 0–4096. When the velocities of the sensing robot were 0 m/s, 0.5 m/s, and 1 m/s, the digital codes were 2751, 2467, and 2270, respectively. These results show that the proposed air flow sensor properly detects the air velocity. The sensitivity of the proposed readout IC was 481 LSB/(m/s).

The minimum detectable air velocity can be calculated as below. The measured root-mean-square (RMS) noise with the integration bandwidth from 0.5 Hz to 200 Hz was 149.9 nV_RMS_, as shown in Figure 16. The gain of the amplifier was set to the maximum gain of 47.47 dB. The RMS output noise was calculated to be 35.4 μV_RMS_. The peak-to-peak output noise could be calculated to be 234 μV_PP_ with typical conversion factor from RMS to peak-to-peak noise of 6.6. This output noise could be converted to 0.29 LSB with the 12-bit ADC with 3.3 V input range. Thus the minimum detectable air velocity could be calculated to be 0.6 mm/s (=234 μV_PP_/(481 LSB/(m/s))).

**Figure 19:**
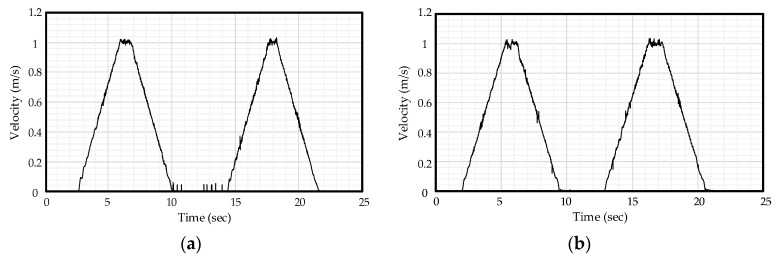
(**a**) Velocity measurement result of Vicon; (**b**) velocity measurement result of proposed readout IC converted from digital codes to analog signal.

Figure 19a presents the air flow velocity measurement result of the Vicons and Figure 19b presents the air flow velocity measurement result of the proposed readout IC converted from digital codes to analog signal. Comparing these two graphs, the proposed readout IC shows accurate measurement of air flow.

Table 1 summarizes the performance of the proposed four-channel low-noise readout IC with those of previous studies [20,26,27,28]. The proposed readout IC has a very wide range of input signals because it has an adjustable gain. In addition, it can be verified that low-noise characteristics are achieved, although the device consumes more current than those of other studies.

## 4. Discussion and Conclusions

This study proposes a micro anemometer air flow sensor and a four-channel low-noise readout IC. The micro anemometer air flow sensors convert temperature changes into resistance changes, and the readout IC converts them into digital signals. The proposed readout IC comprises a current generator in order to heat the micro anemometer of the air flow sensor, three op amp IA for amplification, 12-bit SAR ADC for digital signal conversion, 4th-order LPF, LDO regulator, and SPI. Furthermore, a binary search AOCL is contained to automatically calibrate the offset of the input stage. Because the resistance change in the air flow sensor is tiny, it is important to amplify it with low-noise characteristics. For this purpose, a chopper was applied to the input and output stages of the three op amp IA. In addition, the reason for tuning the current generator is that a typical sensor uses a 100 Ω resistor, but there are large sensor-to-sensor variations. The most offset variation is cancelled out by the automatic offset cancellation loop. To compensate for the gain variations, both the current generator and the instrumentation amplifier are designed to be fully programmable. Moreover, an SPI for communication and an LDO regulator for providing a supply voltage to the digital circuits were integrated into one chip.

The proposed readout IC was fabricated by implementing a 0.18-μm 1P6M CMOS process with an active area of 6.78 mm2. The proposed readout IC consumed 1.886 mA of current at a 3.3 V supply voltage and had a gain range of 4 dB to 48 dB. Because the gain can be adjusted over a wide range, the input voltage range also had a wide range of 0 V to 2 V. The IRN measurement results of the proposed readout IC show that the IRN had a relatively low IRN of approximately 95.4 nV/√Hz at 1 Hz and 10.6 nV/√Hz at 100 Hz. The supply current of the amplifier was 387 uA. Because the thermal noise is generally in inverse proportion to the square-root of the current consumption, the amplifier current is designed to be larger than the previous works. The current consumption of the amplifier is also increased in order to drive the small resistive load for wide-range gain programmability.

These results indicate that the readout IC proposed in this paper is suitable for an air flow sensor using a micro anemometer.

In the research, the micro anemometer sensor and readout IC for the measuring airflow is implemented on PCB. The performances of the presented air flow sensor system are evaluated using the Vicon position measuring system.

## Figures and Tables

**Table 1:** Performance summary between the proposed readout IC and previous studies.

	This Work	TCAS-I 2017 [20]	TIM 2014 [26]	JSSC 2012 [27]	TCAS-I 2004 [28]
Process(μm)	0.18	0.065	0.18	0.7	0.5
Techniques for IA	Chopping	Correlated double sampling	Chopping	Chopping	Conventional
Output format	Digital codes	Voltage	Voltage	Digital codes	Voltage
Supply voltage (V)	3.3	1	2.7	5	2.5
Supply current (μA)	1886 (Full-chip including heater) 387 (IA)	12.3	27.65	270	61
Gain (dB)	4–48	100	40	40	20
Input range	0–2 V	±9.38 mV	±8.8 mV	±40 mV	-
Input referred noise (nV/√Hz)	95.4 (@ 1 Hz) 10.6 (@ 100 Hz)	347.85	84.08	16.2	175

## Data Availability

Not applicable.
